# Resource availability and repeated defoliation mediate compensatory growth in trembling aspen (*Populus tremuloides*) seedlings

**DOI:** 10.7717/peerj.491

**Published:** 2014-07-15

**Authors:** Nadir Erbilgin, David A. Galvez, Bin Zhang, Ahmed Najar

**Affiliations:** Department of Renewable Resources, University of Alberta, Edmonton, Alberta, Canada

**Keywords:** Carbon sink:source relationships, Compensatory responses, Woody plants, Defoliation intensity and frequency, Populus tremuloides, Non-structural carbohydrates

## Abstract

Plant ecologists have debated the mechanisms used by plants to cope with the impact of herbivore damage. While plant resistance mechanisms have received much attention, plant compensatory growth as a type of plant tolerance mechanisms has been less studied. We conducted a greenhouse experiment to evaluate compensatory growth for trembling aspen (*Populus tremuloides*) seedlings under varying intensities and frequencies of simulated defoliation, with or without nutrient enriched media. For the purpose of this study, changes in biomass production and non-structural carbohydrate concentrations (NSC) of roots and leaves were considered compensatory responses. All defoliated seedlings showed biomass accumulation under low defoliation intensity and frequency, regardless of resource availability; however, as defoliation intensity and frequency increased, compensatory growth of seedlings was altered depending on resource availability. Seedlings in a resource-rich environment showed complete compensation, in contrast responses ranged from undercompensation to complete compensation in a resource-limited environment. Furthermore, at the highest defoliation intensity and frequency, NSC concentrations in leaves and roots were similar between defoliated and non-defoliated seedlings in a resource-rich environment; in contrast, defoliated seedlings with limited resources sustained the most biomass loss, had lower amounts of stored NSC. Using these results, we developed a new predictive framework incorporating the interactions between frequency and intensity of defoliation and resource availability as modulators of plant compensatory responses.

## Introduction

Partial or complete defoliation by herbivores is a common event in the life history of most plant species (Cyr & Pace, 1993). Sudden reductions in leaf surface area, or stem and branch tissues due to herbivory can have negative impacts on overall plant fitness, such as reduced growth or reproduction (Bergelson, Juergen & Crawley, 1996; Anten & Ackerly, 2001; Goheen et al., 2007). However, plants have developed two distinctive response mechanisms to cope with herbivore damage: resistance mechanisms, which include any plant trait that prevents or reduces the amount of herbivore damage, and tolerance mechanisms, which include the degree to which plant fitness is altered following damage, relative to the fitness of undamaged plants (Karban & Baldwin, 1997; Strauss & Agrawal, 1999; Tiffin, 2000; Fornoni, 2011). Both mechanisms have been explored as herbivory-coping strategies (Weis & Franks, 2006; Nuñez-Farfán, Fornoni & Valverde, 2007; Stevens, Kruger & Lindroth, 2008; Fornoni, 2011; Lindroth & St Clair, 2013). Although plant resistance mechanisms have been studied extensively, the mechanisms and evolutionary implications of plant tolerance remain one of the major challenges in plant biology and have been received less attention (Tiffin, 2000; Wise & Abrahamson, 2007; Wise & Abrahamson, 2008; Fornoni, 2011). Tolerance mechanisms may include utilization of reserves stored in various plant organs, such as roots and stems, increased photosynthetic activity, compensatory growth, and activation of dormant meristems (Tiffin, 2000). These traits help plants recover from damage through increased production of shoots (King, Eckhart & Mohl, 2008), leaves (Baker et al., 2005), and/or flowers and fruits (Wise, Cummins & De Young, 2008).

Compensatory growth is viewed as a positive plant response and may result in increased biomass or fitness that does not usually occur in the absence of defoliation (McNaughton, 1983; Nowak & Caldwell, 1984; Paige & Whitham, 1987; Trumble, Kolodny-Hirsch & Ting, 1993; Tiffin, 2000; Wise & Abrahamson, 2005; Wise & Abrahamson, 2007; Stevens, Kruger & Lindroth, 2008; Poveda, Jiménez & Kessler, 2010; Fornoni, 2011). Compensatory growth encompasses the range of growth responses to herbivory from overcompensation (i.e., higher growth in damaged than undamaged plants) to complete compensation (i.e., no difference in growth between damaged and undamaged plants) (Belsky, 1986). Tolerance mechanism is common in woody plants (Haukioja & Koricheva, 2000), including deciduous (Lovelock, Pozada & Winter, 1999; Lindroth & St Clair, 2013) and conifers (Bast & Reader, 2003; Zou et al., 2005).

The capacity of a plant to compensate for herbivore damage is often mediated by the strength of resource limitation and carbohydrate sink:source dynamics (Thomson et al., 2003; Stevens, Kruger & Lindroth, 2008). Simply, compensatory mechanisms following damage are mediated by some form of plant growth that alters production and/or reallocation of carbohydrates throughout the whole plant. Growing plant tissues, particularly leaves, flowers, and fruits, generate a strong demand for photoassimilates, becoming sinks for newly synthesized carbohydrates. The strength of this demand can, in turn, regulate the photosynthetic activity of the source leaves (Kaitaniemi & Honkanen, 1996) such that an increase in the number or strength of the sinks can stimulate photosynthesis (Bazzaz et al., 1987). Plant reserves of non-structural carbohydrates (NSC), e.g., starch and soluble sugars, also play an important role in sink:source dynamics. When photosynthetic tissue is damaged or removed by herbivores, especially after repeated, severe defoliations, the remaining foliar tissue is unable to produce enough photoassimilates to fully supply the demand generated by new tissue growth, which is thus supported by the translocation of NSC from other plant organs, such as roots (Tiffin, 2000; Babst et al., 2008; Donaldson, Kruger & Lindroth, 2006; Najar et al., 2014). If these reserves are utilized and depleted, i.e., after repeated severe defoliations, before new photosynthetic tissue is fully functional plant growth and survivorship could be compromised even in resource-rich environments (Landhäusser & Lieffers, 2002; Stevens, Waller & Lindroth, 2007; Stevens, Kruger & Lindroth, 2008; Zhao, Chen & Lin, 2008).

Currently available models predict varied outcomes in plant responses to herbivore damage and resource availability, including increased tolerance in high-resource conditions (compensatory continuum hypothesis, Maschinski & Whitham, 1989), and increased tolerance in limited-resource conditions (growth rate model, Hilbert et al., 1981). Finally, a more dynamic model predicts altered plant tolerance depending on whether the environmental difference represents a limiting resource and whether herbivore damage affects the acquisition of that resource (limiting resource model, Wise & Abrahamson, 2005). A recent literature review suggests that the limiting resource model has a stronger predictive ability than the other more general models (Wise & Abrahamson, 2007). Although proposed mechanisms described in these models may, in some cases, fully account for compensation, plant compensatory responses are highly variable (Banta, Stevens & Pigliucci, 2010; Bagchi & Ritchie, 2011). Furthermore, we do not have a clear understanding of cumulative impacts of repeated defoliations on plant compensatory growth responses over time, nor of how plant compensatory growth responses to defoliation change with subsequent attacks (Massad, 2013).

The objectives of this study were to: (1) characterize the magnitude of compensatory growth for trembling aspen (*Populus tremuloides* (Mich.)) seedlings under varying intensities and frequencies of simulated defoliations, with or without nutrient enriched media in a greenhouse, (2) explore the intricate relationship amongst resource availability, carbon sink:source relationship, and compensatory growth following defoliation, and (3) contextualize our results in a new framework that integrates frequency and intensity of defoliation based on how resource availability may modulate plant compensatory growth. This new framework is intended to provide a range of possible outcomes concerning plant responses to different defoliation pressure under varying resources and to supplement previously published models. For the purpose of this study, changes in biomass production and NSC concentrations of roots and leaves were considered compensatory responses.

## Materials and Methods

### Plant material

Aspen seedlings (*N* = 126) were established from seeds collected near Edmonton (Alberta) (53°32′N 113°30′W). Seedlings were grown for 10 wks under well-watered conditions in a greenhouse at the University of Alberta, and then transplanted into individual 4 L plastic pots, filled with Metromix media (Metro Mix 290, Terra Lite 2000; W. R. Grace of Canada, Ajax, ON, Canada). Pots had four equidistant perforations at the base to allow run out of excess water.

### Study design

After growing for 10 wks under an 18-h photoperiod at 21 °C and with daily watering, seedlings were randomly divided into three groups of 42 plants, and each group was assigned to one of three defoliation intensities: low (25% reduction in leaf area), high (75% reduction in leaf area), and non-defoliated controls (Table 1). The intensity and frequency of the defoliation treatments were designed to approximate insect defoliation levels during non-outbreak (low defoliation) and outbreak (high defoliation) population densities (e.g., Parry, Herms & Mattson, 2003). Further, manual defoliation enabled us to control the amount and timing of damage.

**Table 1:** Annotated experimental design. Annotated experimental design evaluating how carbon sink–source relationships and compensatory plant growth operate under different intensities and frequencies of simulated defoliation of aspen seedlings, with or without fertilizer treatments. Number of replicates is 7 for all treatments. No harvest was performed after the first defoliation.

**Intensity level**	Control (0% reduction) 1						LOW (25% reduction in leaf area)						HIGH (75% reduction in leaf area)					
Frequency	–		–		–		2		3		4		2		3		4	
Fertilization	F	NF	F	NF	F	NF	F	NF	F	NF	F	NF	F	NF	F	NF	F	NF
**Harvest and defoliation schedule**																		
1st D May 12–13	**–**	**–**	**–**	**–**	**–**	**–**	**D**	**D**	**D**	**D**	**D**	**D**	**D**	**D**	**D**	**D**	**D**	**D**
2nd D May 26–27	**–**	**–**	**–**	**–**	**–**	**–**	**D**	**D**	**D**	**D**	**D**	**D**	**D**	**D**	**D**	**D**	**D**	**D**
3rd D & 1st H June 9–10	**H**	**H**	**–**	**–**	**–**	**–**	**H**	**H**	**D**	**D**	**D**	**D**	**H**	**H**	**D**	**D**	**D**	**D**
4th D & 2nd H June 23–24			**H**	**H**	**–**	**–**			**H**	**H**	**D**	**D**			**H**	**H**	**D**	**D**
3rd H July 7–8					**H**	**H**					**H**	**H**					**H**	**H**

**Notes.**

Experimental treatments F Fertilized NF Not fertilized

Experimental tasks D Defoliation H Harvest

Seedlings in low or high defoliation groups were defoliated with scissors by cutting the distal section of all leaf blades corresponding to the defoliation treatments described above. After initial defoliation treatments were applied, 21 seedlings from each group were randomly selected and fertilized biweekly with a complete N:P:K fertilizer (15:30:15) dissolved in water until the end of the experiment (Table 1). To explore the effect of defoliation frequency, the same defoliation protocol was repeated every 2 wks two, three or four times in the remaining seedlings. Two weeks after the second defoliation was applied, one third of the seedlings in each treatment group were harvested, immediately before the third defoliation was applied. We repeated a similar harvesting schedule before the third and fourth defoliation treatments until all seedlings were harvested (Table 1). Harvesting 2 wks after each defoliation allowed us to capture the effect of the previous defoliation on biomass and NSC allocation. Immediately following harvest, leaves and roots (soil medium was rinsed from the roots) were separated and oven-dried at 70 °C for 48 h, and weighed to estimate dry biomass.

Total NSC concentrations of leaves and roots were measured as described by Chow & Landhäusser (2004). Briefly, after drying the root and leaf tissues, 5 g of leaves or roots from all seedlings were ground in a Wiley Mill to pass through 40 mesh. Soluble sugars were extracted three times with hot 80% ethanol solution, followed by a reaction between the extract and phenol–sulfuric acid which allowed sugars to be measured colourimetrically. To measure starch concentration, the tissue remaining following ethanol extraction was digested with the enzymes *α*-amylase (ICN 190151, from *Bacillus lichenformis*) and amyloglucosidase (Sigma A3514, from *Aspergillus niger*) followed by a colourimetrically-measurable reaction with peroxidase-glucose oxidase-o-dianisidine (Sigma Glucose Diagnostic Kit 510A). Total NSC were the sum of water soluble sugars and starch. We summed individual sugar and starch in our study as the combined total provides a more accurate reflection of what herbivores ingest during defoliation.

### Data analysis

Significant differences in leaf and root dry weights, and NSC concentrations between treatments were found using three-way ANOVAs (Fertilization × Defoliation intensity × Defoliation frequency) according with the General Linear Model. In all cases, normality and equal variance tests were performed to verify that ANOVA requirements were satisfied. When statistical differences were detected, pairwise multiple comparison procedures (Holm-Sidak method) were performed between and within treatments and treatment levels. All procedures were performed with the statistical software SigmaStat 4 (Systat Software Inc, Chicago, IL).

## Results

### Leaf biomass

Overall, leaf dry biomass of aspen seedlings varied depending on fertilization and defoliation frequency (*F* = 5.83, *P* = 0.02; *F* = 29.99, *P* < 0.001, respectively) and showed a similar pattern at both low and high intensities of defoliations. We also detected a significant interaction between intensity and frequency of defoliation on leaf dry biomass (*F* = 2.72, *P* = 0.03).

**Figure 1:**
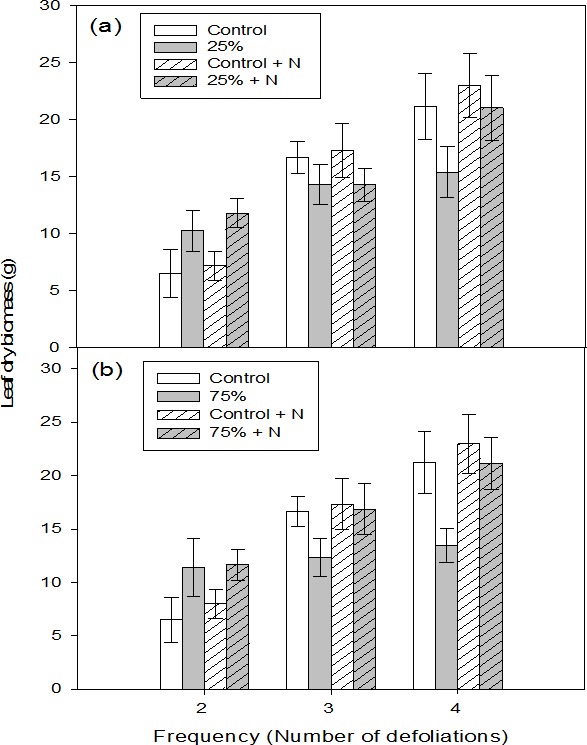
Leaf dry biomass of aspen seedlings after low (A, 25%) and high (B, 75%) intensity of defoliations, repeated two, three, and four times. Hatched bars indicate fertilized treatments. Each bar and error bar gives the mean and SE, respectively, for seven seedlings.

At low intensity defoliation, only defoliation frequency had a significant effect on leaf dry biomass and the interaction between fertilization and defoliation frequency was also significant (Table 2). After two repeated defoliations, the effect of defoliation on leaf dry biomass was positive and leaf dry biomass of defoliated seedlings, whether they were fertilized or not, was higher than that of fertilized or unfertilized control seedlings (Table 3, Fig. 1A). In contrast, leaf dry biomass was similar among seedlings in all four treatment categories after three repeated defoliations (Table 3, Fig. 1A). Furthermore, unfertilized seedlings defoliated four times had the lowest biomass relative to those in the remaining three treatment categories (Table 3, Fig. 1A).

**Table 2:** Summary of statistical analysis results for experiments testing leaf and root biomass and non-structural carbohydrate (NSC) concentrations of leaves and roots. In response to fertilization and defoliation frequency under each defoliation intensity.

Source	Low intensity (<25% defoliation)				High intensity (>75% defoliation)			
	Leaf (F-ratio and P-value)		Root (F-ratio and P-value)		Leaf (F-ratio and P-value)		Root (F-ratio and P-value)	
	Biomass	NSC	Biomass	NSC	Biomass	NSC	Biomass	NSC
Defoliation Frequency (DF)	25.99^**^	3.78^*^	13.52^***^	27.25^***^	9.62^*^	∼	12.64^**^	29.34^***^
Fertilization (F)	∼	36.9^***^	17.89^***^	42.13^***^	6.56^*^	28.68^***^	15.78^***^	50.89^***^
DF × F	14.16^***^	10.71^**^	16.23^***^	13.97^***^	13.32^***^	∼	13.85^***^	15.48^***^

**Notes.**

Fixed factors are tested with F-test statistics. Only significant interactions are reported, with main effects or their interactions marked ∼ removed because they were not significant.

Significance is given as:

*** *P* < 0.0001.

** *P* < 0.001.

* *P* < 0.05.

**Table 3:** Summary of statistical analysis results for experiments testing leaf and root biomass and non-structural carbohydrate (NSC) concentrations of leaves and roots. At different defoliation frequencies under each defoliation intensity.

Defoliation frequency	Low intensity (<25% defoliation)				High intensity (>75% defoliation)			
	Leaf (F-ratio & P-value)		Root (F-ratio & P-value)		Leaf (F-ratio & P-value)		Root (F-ratio & P-value)	
	Biomass	NSC	Biomass	NSC	Biomass	NSC	Biomass	NSC
Two defoliations	5.66^**^	∼	∼	5.46^**^	6.31^**^	6.79^**^	∼	6.23^**^
Three defoliations	∼	8.12^***^	4.78^*^	9.34^***^	4.99^*^	6.18^**^	6.24^**^	9.87^***^
Four defoliations	4.85^*^	8.97^***^	7.37^**^	8.97^***^	5.78^**^	6.54^***^	11.75^***^	10.12^***^

**Notes.**

Fixed factors are tested with F-test statistics. Only significant interactions are reported, with main effects or their interactions marked ∼ removed because they were not significant.

Significance is given as:

*** *P* < 0.0001.

** *P* < 0.001.

* *P* < 0.05.

At high intensity defoliation, both defoliation frequency and fertilization had significant effects on leaf biomass and the interaction between these two was also significant (Table 2). At the lowest defoliation frequency, defoliation had a positive effect on leaf biomass and seedlings defoliated two times had higher biomass than control seedlings, regardless of fertilizer application (Table 3, Fig. 1B). In contrast, as the defoliation frequency increased, unfertilized seedlings had the lowest biomass compared to the seedlings in the remaining three categories (Table 3, Fig. 1B). In particular, the reduction in leaf dry biomass was more apparent at the highest defoliation frequency where biomass was decreased by about 60% compared to defoliated and fertilized seedlings.

### Root biomass

Overall, root dry biomass was influenced by defoliation frequency (*F* = 51.19, *P* < 0.001) and there was a significant interaction between fertilization and defoliation intensity (*F* = 3.14, *P* = 0.05). Between low and high intensity defoliation, root biomass showed a similar pattern to leaf dry biomass.

**Figure 2:**
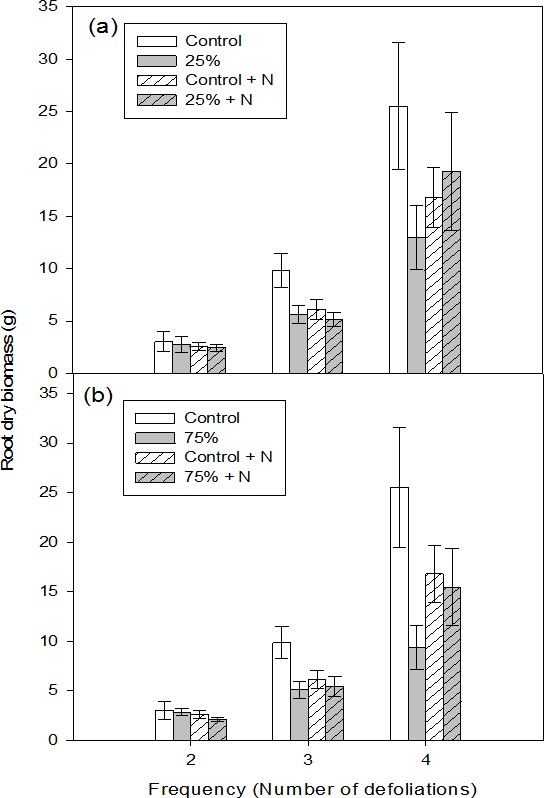
Root dry biomass of aspen seedlings after low (A, 25%) and high (B, 75%) intensities of defoliations, applied two, three, and four times. Hatched bars indicate fertilized treatments. Each bar and error bar gives the mean and SE, respectively, for seven seedlings.

At low intensity defoliation, the overall interaction between fertilization and defoliation frequency was significant and there was a significant reduction in root biomass of fertilized, but not defoliated seedlings (Table 2). After two repeated defoliations, root dry biomass was similar among all seedlings (Table 3, Fig. 2A). However, after three repeated defoliations, root dry biomass of seedlings in the unfertilized control treatment was almost twice that of unfertilized-defoliated seedlings regardless of defoliation intensity (Table 3, Fig. 2A). After four low intensity defoliation, unfertilized control seedlings produced a two-fold increase in root dry biomass in comparison with unfertilized defoliated seedlings (Table 3, Fig. 2A).

At high intensity, defoliation frequency, fertilization and their interactions were significant (Table 2). There was no difference in root dry biomass after two repeated defoliations (Table 3, Fig. 2B). After three defoliations, unfertilized control seedlings had more root biomass than seedlings in the remaining three treatment categories (Table 3, Fig. 2B). Notably, after the fourth defoliation, unfertilized-defoliated seedlings had the lowest root biomass and the control unfertilized seedlings had the highest (Table 3, Fig. 2B).

### Non-structural carbohydrate (NSC) concentration of leaves

Overall, fertilization had a significant effect on leaf NSC concentration (*F* = 54.92, *P* < 0.0001). The interaction between fertilization and defoliation frequency also influenced leaf NSC concentrations (*F* = 12.54, *P* < 0.0001). However, leaf NSC concentration of fertilized seedlings was not influenced by defoliation intensity or frequency.

**Figure 3:**
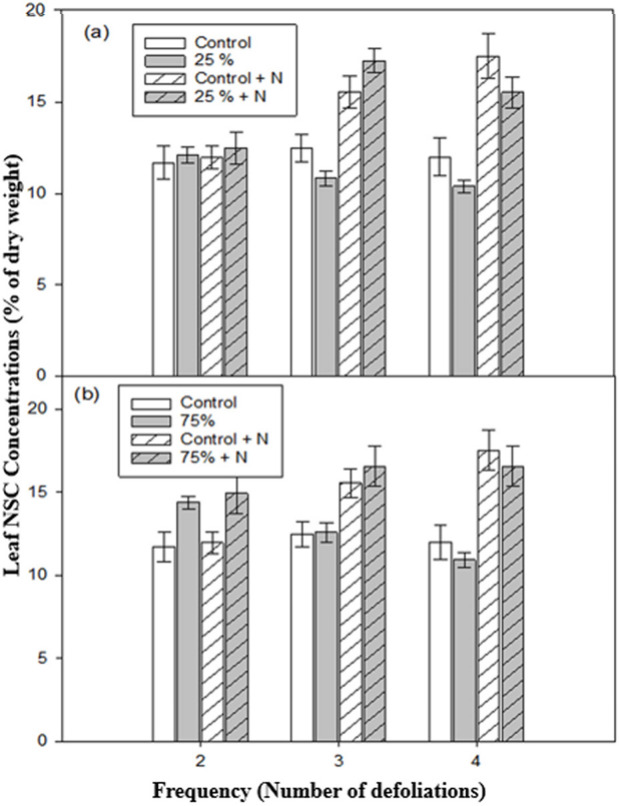
Leaf non-structural carbohydrate (NSC) concentrations of aspen seedlings after low (A, 25%) and high (B, 75%) intensities of defoliation, applied two, three, and four times. Hatched bars indicate fertilized treatments. Each bar and error bar gives the mean and SE, respectively, for seven seedlings.

At low intensity defoliation, defoliation frequency and fertilization had significant effects on leaf NSC concentration and their interaction was also significant (Table 2). Although leaf NSC concentration was similar among seedlings after two defoliations (Table 3, Fig. 3A), when defoliation frequency increased to three or four times, fertilized seedlings in control or defoliated categories had higher leaf NSC concentration than unfertilized seedlings (Table 3; Fig. 3A). The leaf NSC concentration was not different between control and defoliated seedlings, at all defoliation intensities. At both frequency levels, unfertilized-defoliated seedlings had the lowest leaf NSC concentration.

At high intensity, overall leaf NSC concentration varied only with fertilization (Table 2). After two repeated defoliations, NSC concentration of defoliated seedlings was higher than that of non-defoliated seedlings, regardless of fertilizer treatment (Table 3, Fig. 3B). However, after three and four repeated defoliations, fertilized seedlings had a higher leaf NSC concentration than unfertilized seedlings, and there was no difference between fertilized-defoliated and fertilized-control seedlings or between unfertilized-defoliated and unfertilized-control seedlings (Table 3, Fig. 3B).

### Non-structural carbohydrate (NSC) concentration of roots

Overall, both fertilization and defoliation frequency, and their interactions, significantly influenced root NSC concentrations (Table 2). In general, fertilized seedlings had higher amounts of NSC in their roots than unfertilized seedlings in all defoliation intensities and frequencies.

**Figure 4:**
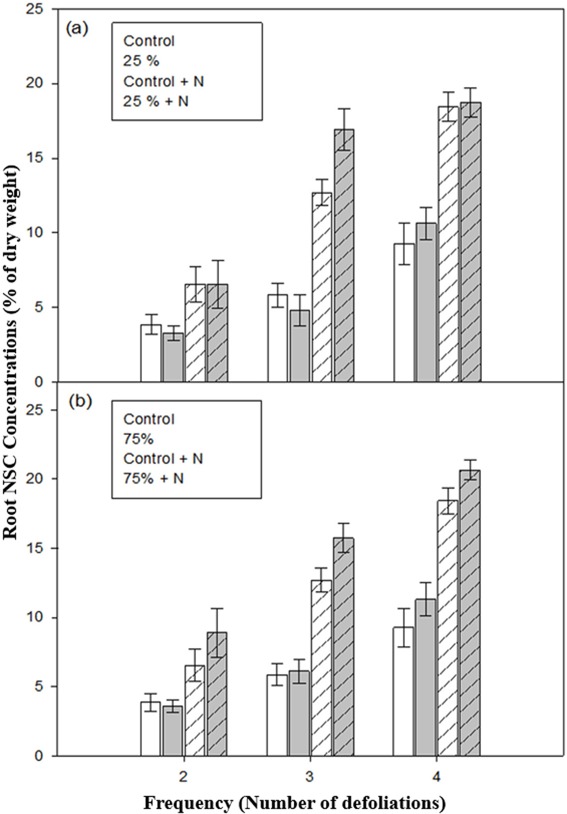
Root non-structural carbohydrate (NSC) concentrations of aspen seedlings after low (A, 25%) and high (B, 75%) intensities of defoliations, applied two, three, and four times. Hatched bars indicate fertilized treatments. Each bar and error bar gives the mean and SE, respectively, for seven seedlings.

At low intensity defoliation, defoliation frequency and fertilization had significant effects on root NSC concentration and their interaction was also significant (Table 2, Fig. 4A). After two or four repeated defoliations, fertilized defoliated or control seedlings had higher amounts of NSC than those without fertilization (Table 3, Fig. 4A). Root NSC concentration was similar between fertilized-defoliated and fertilized-control seedlings, and between unfertilized-defoliated and unfertilized-control seedlings. Seedlings fertilized and defoliated three times had the highest root NSC concentrations compared to those in the remaining three treatments (Table 3, Fig. 4A).

At high intensity defoliation, defoliation frequency and fertilization had significant effects on the amount of NSC in roots, and their interaction was also significant (Table 2). Once again, fertilized seedlings had a higher root NSC concentration than unfertilized seedlings (Table 3, Fig. 4B). Further, seedlings fertilized and defoliated three or four times had the highest root NSC concentrations among the remaining seedling treatments.

**Figure 5:**
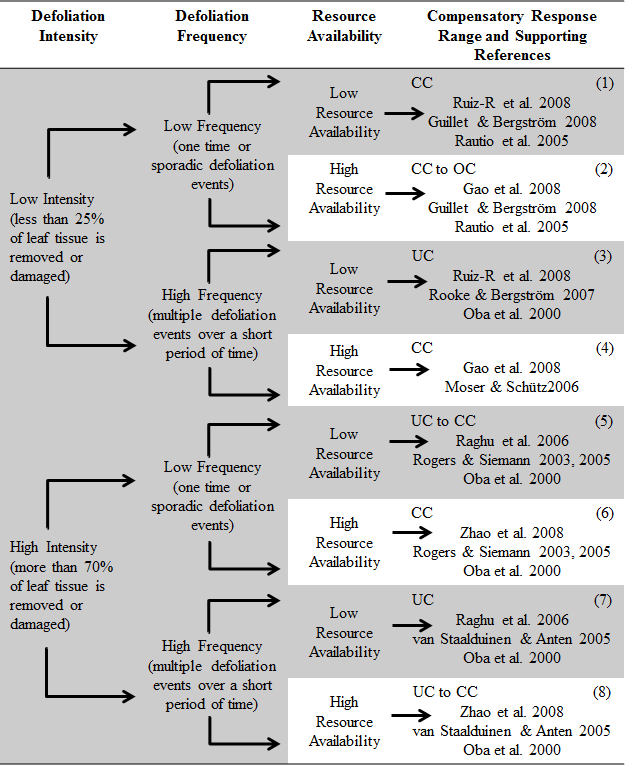
Conceptual diagram of the proposed framework. The framework predicts a range of compensatory plant responses (biomass only) including undercompensation (UC), complete compensation (CC) and overcompensation (OC) following defoliation, depending on the intensity and frequency of defoliation and resource availability for plant recovery. Examples of peer-reviewed articles supporting the framework predictions are provided for each prediction range.

Based on these results and the results of earlier studies, we developed a new framework shown in Fig. 5, which provides the summary of outcomes in plant compensatory growth under varying degrees of herbivory pressure and resource availability. Its parameters and explanations are provided in the discussion section.

## Discussion

Plant responses to repeated intensive defoliation are seldom studied (Tiffin, 2000; del-Val & Crawley, 2005; Stevens, Kruger & Lindroth, 2008). In the current study, aspen seedlings showed complete biomass accumulation under low defoliation intensity and frequency, regardless of resource availability. However, as defoliation intensity and frequency increased, compensatory responses of seedlings altered depending on fertilizer application. Aspen in a resource rich environment showed complete compensation, but responses ranged from undercompensation to complete compensation in a resource limited environment. Furthermore, when plants receive sufficient fertilization, they primarily allocated resources for growth. In contrast, when they received insufficient or no resources, below ground portion of plants received priority in resource allocation, i.e., increasing root biomass, while above ground portion suffered from lack of resources, i.e., biomass reduction. Overall, these results are in agreement with previous reports (e.g., McPherson & Williams, 1998; del-Val & Crawley, 2005) and provide much needed information on compensatory responses in plants under intensive and frequent defoliation (Tiffin, 2000; Wise & Abrahamson, 2008). These results demonstrate that up to a certain threshold, defoliation is not detrimental to plants, but beyond this point, defoliation can harm plants (Fornoni & Nuñez-Farfán, 2000; del-Val & Crawley, 2005). In the current study, this threshold was driven by the defoliation frequency and resource availability. Since foliage and roots play distinctive roles in plant compensatory growth, we discussed their individual contributions to the compensatory mechanisms in aspen.

### Leaf biomass accumulation in relation to varying resource availability and defoliation

The impact of defoliation on leaf biomass was mediated by the interaction between intensity and frequency of defoliation as well as by resource availability. After two high-intensity defoliations, plant growth resulted in complete compensation (i.e., leaf biomass was same between defoliated and non-defoliated plants, regardless of resource availability), and in overcompensation (i.e., leaf biomass was higher in defoliated than in non-defoliated plants, regardless of fertilizer application) after two low-intensity defoliations. These results demonstrate that defoliation frequency plays a more critical role in modulating compensatory growth at a low intensity of defoliation than resource availability. The less critical role of resource availability as a modulator of compensatory growth under low defoliation frequency is also supported by the lack of differences between fertilized and unfertilized defoliated plants.

The effect of resource availability on compensatory growth gradually increased with increasing frequency and intensity of defoliations. After three low-intensity defoliations, compensatory growth still resulted in non-significant differences between defoliated and control plants, regardless of fertilizer treatment. However, after three or more high-intensity defoliations, only fertilized plants completely compensated for defoliation and unfertilized plants lost significant leaf biomass. These results show that at high-intensity defoliation, resource availability plays a more critical role in modulating compensatory growth than defoliation frequency, suggesting that a trade-off in allocation of resources between sinks and sources was manifested only after repeated defoliations. These results are consistent with earlier results (Wise & Abrahamson, 2008); in 12 of 18 studies reviewed, compensatory growth occurred only in plants in resource-rich environments. Overall, the interaction between defoliation and resource availability determines the patterns and magnitudes of biomass accumulation in foliage, as mechanisms of plant tolerance to defoliation.

Although we did not measure, higher leaf biomass suggests increased photosynthetic rates of leaves remaining on after defoliation to compensate for growth loss (Thomson et al., 2003; Gálvez & Cohen-Fernández, 2006). For instance, Stevens, Kruger & Lindroth (2008) reported a 22–32% increase in photosynthetic rates in defoliated and fertilized aspen seedlings, although in this particular study there was no relationship between plant tolerance and compensatory photosynthetic rates. Differences between this study and Stevens, Kruger & Lindroth (2008) may be attributed to the methods used for seedling establishment as the earlier study used clones which may have exhibited genetic constraints on plant tolerance whereas our seedlings were established from seeds that may have not shown such genetic constraints as they came from different genetic background.

### Root biomass accumulation in relation to varying resource availability and defoliation

While compensatory growth does not occur in non-damaged roots, the large changes in the sink:source relationship experienced by defoliated seedlings may have been important in driving some of the differences observed in root biomass accumulation among treatments. We found that after three or more repeated low- or high-intensity defoliations, root biomass was higher in non-fertilized control than in fertilized defoliated or control seedlings. This result is not unexpected as fertilized aspen seedlings prioritize allocation to leaves or shoots over roots (Landhäusser & Lieffers, 2002; Stevens, Kruger & Lindroth, 2008). We interpreted the large differences in root biomass between control and defoliated seedlings in a resource limited environment as a result of two main co-occurring processes: (a) after defoliation, translocation of photoassimilates from foliage to roots is severely reduced, which in turn reduces root growth, and (b) as new stem buds are activated to produce new leaves, roots translocate reserves to promote active growth in these carbohydrate sinks, which further compromises root growth. These predictions are based on the assumption that defoliation alters resource allocation in woody plants (Babst et al., 2008; Stevens, Kruger & Lindroth, 2008; Najar et al., 2014) and resources are utilized for growth of remaining plant tissues, particularly leaves (Landhäusser & Lieffers, 2002; Babst et al., 2008; Hochwender et al., 2012). Furthermore, substantial allocation of resources to roots may not be advantageous to early successional tree species–like aspen, because increasing above ground biomass, such as leaves and stems, may improve aspen’s competitive ability to capture lights.

### Plant compensatory responses under varying resource availability and defoliation

In the current study, at the highest defoliation intensity and frequency, NSC concentrations in leaves and roots were similar between fertilized defoliated and fertilized non-defoliated seedlings, demonstrating high sink:source activity driven primarily by herbivore damage (Körner, 2003). This also demonstrates that even under high-defoliation pressure, aspen seedlings parted their resources between growth and reserves. In contrast, defoliated plants with limited access to resources sustained the most biomass loss and had lower amounts of stored NSC, showing reduced sink:source activity (Körner, 2003). This may also indicate that both growth and reserves suffer from resource limitation. In parallel, earlier studies similarly reported that defoliation can influence the mobilization and storage of NSC between sinks and sources depending on resource availability (Babst et al., 2008; Schwachtje et al., 2005; Hochwender et al., 2012). However, our results contradict the results of Rivera-Solís et al. (2012) who found higher carbon accumulation in roots of defoliated *Ruellia nudiflora*–a perennial herb–relative to non-defoliated plants. It is likely that in the current study carbon accumulated in roots of defoliated and fertilized seedlings was depleted shortly after defoliation, as the time between each defoliation-harvest schedule was two weeks and may not have been long enough to fully restore reserves (Eyles, Pinkard & Mohammed, 2009); the time between each defoliation-harvest was one month in the earlier study, where reserves were likely re-accumulated once the new ‘source’ tissues were fully functional. Our interpretations about the role of NSC in sink–source dynamics above should be viewed caution as stems are also used by aspen to store reserves. Since we did not measure NSC concentrations in stems, we do not know how this could affect our conclusions. However, regardless of study systems, reserves can potentially serve to compensate for herbivore damage in plants (Babst et al., 2008; Schwachtje et al., 2005; Stevens, Kruger & Lindroth, 2008; Hochwender et al., 2012).

### Suggested framework for future studies

Despite the relatively recent increase in studies on plant compensatory responses, our empirical knowledge of these responses, especially the consequences of repeated defoliations on a plant’s ability to compensate damage, is still limited. Although our work is focused on compensatory growth in aspen, understanding the mechanisms that drive variation in compensation leads to predictive insight into how plants in general cope with defoliation. Here, we synthesized how resource availability combined with defoliation intensity and frequency affects the plant’s compensatory responses in a new framework. This framework integrates interactions between biotic and abiotic factors into the framework of plant compensatory responses, particularly in woody plants, as plant responses to defoliation can be different between trees and herbs (Haukioja & Koricheva, 2000; Muola et al., 2010; Barton, 2013).

Although the framework was developed based on leaf damage, our conclusions may also be applied to research focused on apical meristem damage, which elicits similar modifications in patterns of resource allocation to various plant organs (Stowe et al., 2000). Likewise, plants generally exhibit compensatory growth in response to damage on the apical meristem as on the foliage (Trumble, Kolodny-Hirsch & Ting, 1993) and plant compensatory responses are similarly subjected to biotic (damage intensity and frequency) and abiotic (resource availability) limitations. Plant compensatory responses are also modulated by plants’ intrinsic factors, such as phenology, pre-herbivory reserve states, or ontogeny (Maschinski & Whitham, 1989; Stowe et al., 2000; Tiffin, 2000; Gruntman & Novoplansky, 2011).

This new framework was built on three core conjectures: (i) it considers changes in sink:source dynamics and utilization of plant reserves as the driving mechanisms behind our predictions, (ii) its predictions are modulated by interactions between intensity and frequency of defoliation and level of resources available to plants following defoliation, and (iii) its predictions are presented as a range of compensatory responses instead of precise outputs. The new framework assumes that defoliation mostly affects above-ground tissue and that plant reserves are available. It predicts that the overall impact of defoliation on plant growth is altered by interactions among the three factors mentioned above: when defoliation intensity is low (e.g., less than 25% of leaf tissue removed), frequency plays a more important role modulating plant compensatory growth responses than resource availability. If intensity is high (e.g., more than 70% of leaf tissue removed), resource availability is more important in controlling compensatory growth responses than frequency of defoliation. Figure 5 shows our predictions and provides some examples of peer-reviewed literature in agreement with them. We were limited by the low numbers of studies that looked at both intensity and frequency of defoliation and resource availability. Detailed descriptions of these interactions are presented below.

### Compensatory responses under low intensity defoliation

Under this scenario, we predict that, if defoliation frequency is low, the negative impact of defoliation on plant growth is overcome by compensatory mechanisms. Resource limitation is not expected to limit the capacity for recovery following defoliation, and in fact might be advantageous given the higher growth compared to controls (Barry et al., 2011). The production of photoassimilates by remaining photosynthetic tissues is expected to increase at low defoliation levels due to increased light penetration, changes in sink:source dynamics and water relations (i.e., a higher ratio between the area of water absorbing tissue to transpiring leaf tissue). The added impact of one or more of these mechanisms on plant growth may result in complete compensation (output 1, Fig. 5). This same scenario may also result in overcompensatory growth (output 2, Fig. 5) if resource availability and NSC reserves are high.

Defoliation frequency may modulate these outcomes. If defoliation frequency is high, the cumulative cost of replacing the damaged or removed leaf tissue may outweigh the benefits resulting from increase in photosynthetic tissue production even when resource availability is high (Coley, Bryant & Chapin, 1985). This scenario results in undercompensatory growth under low resource availability and depletion of NSC reserves, and complete compensation only under high NSC reserves and resource availability (outputs 3 and 4, Fig. 5).

### Compensatory responses under high intensity defoliation

We predict that when both resource availability and defoliation intensity are high, but defoliation frequency is low, plants completely compensate, but do not overcompensate, for the negative effects of defoliation to maximize fitness (Thomson et al., 2003). This idea is supported by a number of studies reporting compensatory responses in various plant systems (Paige & Whitham, 1987; Wallace & Macko, 1993). We predict that any increase in the photosynthetic rate of remaining leaf tissue resulting from any of the mechanisms previously described may be overcome by a negative feedback cycle in sink:source relationships (Thomson et al., 2003). This negative feedback begins when the small amount of remaining leaf tissue is unable to export enough photoassimilates to maintain root growth. As root growth rate is reduced, water transport to remaining leaf tissue is also reduced. If water transport to leaves is reduced, a cascade of physiological mechanisms is enabled to maintain positive leaf water balance, primarily by reducing stomatal conductance. Finally, if stomata are closed, water balance is maintained but photosynthesis is reduced, which reinforces the negative feedback cycle.

The negative impact of limited water availability on growth and NSC accumulation of aspen seedlings have been extensively studied in recent years (e.g., Gálvez, Landhäusser & Tyree, 2011; Gálvez, Landhäusser & Tyree, 2013). Likewise, Rivera-Solís et al. (2012) suggested that high intensity defoliation alters water allocation among different parts of *R. nudiflora*, and that allocation of water to reproductive structures induces water stress in foliage, which may cause stomatal closure and thus a reduction in photosynthetic rates in foliage. Our model predicts that high intensity, but low frequency, defoliation may result in undercompensation if resource availability is low, and complete compensation if resource availability is high (outputs 5 and 6, Fig. 5). As defoliation frequency increases, plant survivorship will increasingly depend on NSC reserves, which will be translocated to maintain leaf tissue. If defoliation events are of high intensity and frequency, plants are expected to severely undercompensate in environments with low resources. This parallels the results of Barry et al. (2011), where seedlings of *Eucalyptus globulus* showed a reduced ability to recover from repeated defoliations in a resource-limited environment. Conversely, we expect complete compensation if resource availability is high (outputs 7 and 8, Fig. 5), until all NSC reserves in the roots are depleted, which may potentially lead to plant death.

### Limitations

Two main limitations of this study are important to consider in relation to its main findings. First, plant ontogeny will likely affect the efficacy of plant compensatory responses, as young and mature plants differ in their capacity to store resources and in how they acquire and prioritize (i.e., among growth, storage and reproduction) resources (Haukioja & Koricheva, 2000; Muola et al., 2010; Barton, 2013; Erbilgin & Colgan, 2013). However, in a recent meta-analysis on the ontogeny of plant tolerance to herbivory, Barton & Koricheva (2010) found similar expressions of tolerance among seedlings, juveniles and mature stages, although there are individual cases in which tolerance has been shown to increase (Boege et al., 2007) or decrease (Thomson et al., 2003) across ontogeny. Likewise, Massad (2013) found differences in growth responses to herbivore damage between ontogenetic stages in perennial herbs, but not in long-lived trees. Further research is needed to determine the mechanisms that enable juvenile and mature woody plants to tolerate herbivory.

Second, we used mechanical defoliation to control the level and timing of defoliation on seedlings as such a technique may be the only way to control levels of damage and to study compensatory responses in woody plants (Boege, 2005). It is well established that herbivore defoliation can provide a more natural elicitation of plant responses to damage (Karban & Baldwin, 1997; Thomson et al., 2003; Boalt & Lehtilä, 2007), but physical damage has also been shown to elicit modifications in patterns of resource allocation to various vegetative and reproductive organs (e.g., Marshall et al., 2005; Stevens, Kruger & Lindroth, 2008). Using mechanical defoliation in the current study therefore likely did not increase the chances of revealing the positive effects of plant damage. More importantly, the type of herbivore (leaf-chewing, grazing, browsing, etc.) and the location of herbivory (foliage, roots, etc.) on the plant is more important than how damage is induced (Massad, 2013), emphasizing the need for additional studies before generalizations can be made.

## Supplemental Information

10.7717/peerj.491/supp-1 Supplemental Information 1 Non-structural carbohydrates

10.7717/peerj.491/supp-2 Supplemental Information 2 Plant biomass
